# Cost, safety, and rehabilitation of same-stage, bilateral total knee replacements compared to two-stage total knee replacements

**DOI:** 10.1186/s43019-021-00098-z

**Published:** 2021-06-12

**Authors:** Raymond C. W. Wan, Jason C. H. Fan, Yuk-Wah Hung, Ka-Bon Kwok, Carmen K. M. Lo, Kwong-Yin Chung

**Affiliations:** 1 Department of Orthopedics & Traumatology, Prince of Wales Hospital, Sha Tin, Hong Kong SAR, China; 2 Present address: Department of Orthopedics & Traumatology, Alice Ho Miu Ling Nethersole Hospital, Tai Po, Hong Kong SAR, China

**Keywords:** Knee osteoarthritis, Total knee replacement, Safety, Cost, Rehabilitation outcome

## Abstract

**Background:**

Many patients experience bilateral knee osteoarthritis and require bilateral total knee replacement (TKR). Same-stage, bilateral TKR is proposed to be a cost-effective and safe solution compared to two-stage, but conflicting results in the literature are reported. We aim to compare the costs, safety, and rehabilitation performance of patients in same-stage versus two-stage, bilateral TKR with our centre’s perioperative protocol.

**Materials and methods:**

We retrospectively reviewed 175 patients (95 same-stage, 80 two-stage) who had undergone bilateral TKR in our centre. Patient selection for same-stage, bilateral TKR was strictly protocol-driven and required fulfilment of all criteria, including age < 75 years, American Society of Anesthesiologists (ASA) grade 1 or 2, body mass index (BMI) < 40, and having non-complex arthritis. All patients followed a standardised pre-operative, intra-operative, and post-operative Enhanced Recovery After Surgery (ERAS) protocol. The cost, safety profiles, and rehabilitation outcomes were compared between the same-stage and two-stage groups.

**Results:**

The same-stage, bilateral TKR reduced the length of hospital stays by 5.71 days per patient, decreased the operation time by 27.4 min, saved 3.34 (18.6%) physiotherapy sessions, and 3.78 (51.5%) occupational therapy sessions. The same-stage group experienced a higher haemoglobin drop but no significant difference in transfusion percentage, transfusion volume, complication rate, and readmission rate. The two-stage subgroup with anaesthetic risk, age, and BMI similar to the same-stage group showed the same results. Same-stage, bilateral TKR patients experienced no significant difference in final post-operative pain levels and rehabilitation outcomes as two-stage TKR patients.

**Conclusion:**

This study showed that same-stage, bilateral TKR can reduce costs, with similar safety profiles and rehabilitation outcomes compared to the two-stage, bilateral TKR.

## Introduction

Knee osteoarthritis is a prevalent disease globally, that affects many patients. In the United States, 12% of adults are affected by knee osteoarthritis, and total knee replacements (TKRs) cost USD10.2 billion USD annually [1]. Similarly, 8.1% of the Chinese population is affected by symptomatic knee osteoarthritis [2]. Bilateral knee osteoarthritis is common in that as many as 34.1% female and 17.5% male Chinese individuals aged over 65 years suffer from bilateral radiographic knee osteoarthritis [3]. The demand for bilateral TKR is high, leading to a massive healthcare burden. Therefore, a safe and more cost-effective solution is required.

Bilateral knee replacements can be performed either in a same-stage (both knees in the same anaesthetic session) or two-stage (two replacements on two separate operations that can be weeks or months apart) surgical procedure. The advantages of a same-stage, bilateral TKRs include one anaesthetic session, single theatre use, and a single hospital admission, and shorter hospital stay [4–6]. Multiple studies reported that same-stage, bilateral TKR is a safe procedure [2, 7, 8], with similar morbidities and mortality [9]. The disadvantages include the possibility of more significant post-operative pain, increased rate of cardiovascular events, thromboembolism, blood loss, and mortality [3, 10]. In addition, some studies reported lower overall cost to the healthcare system [4, 6], while others showed no overall cost reduction [5, 11]. There are also conflicting reports with regards to rehabilitation outcomes [9, 12]. Some studies are outdated while modern practice may improve outcomes significantly. Overall, the net outcome of same-stage, bilateral TKR—whether it is beneficial to patients in terms of safety and rehabilitation and whether it is beneficial to the healthcare system in terms of cost-effectiveness—remains a controversial topic.

Since 2016, the surgeons at the Joint Replacement Centre (JRC) of Alice Ho Miu Ling Nethersole Hospital in Hong Kong have performed same-stage, bilateral TKR procedures with a standardised protocol of Enhanced Recovery After Surgery (ERAS). This study aims to compare the cost, safety profile, and clinical outcomes of a same-stage, bilateral TKR procedure versus a two-stage, bilateral TKR with patients undergoing the same ERAS protocol. We hypothesized that same-stage, bilateral TKR would reduce cost with similar safety profile and clinical outcomes compared to two-stage, bilateral TKR.

## Materials and methods

### Patient selection protocol and defining the patient cohorts

This retrospective review studied data from all patients with bilateral TKR performed between January 2016 and December 2017 at the JRC in Alice Ho Miu Ling Nethersole Hospital, Hong Kong SAR, China. All patients with bilateral primary TKR performed for bilateral knee osteoarthritis were included. The reviewed patients were segregated into two cohorts: same-stage, bilateral TKR and two-stage, bilateral TKR, according to the surgery that they ultimately received (*as treated*).

In our centre, patient selection for same-stage, bilateral TKR was strictly protocol-driven. For patients undergoing same-stage, bilateral TKR, they had to fulfil all of the following criteria:

- Age < 75 years
- American Society of Anesthesiologists (ASA) class 1 or 2 according to the anaesthetists’ documentation
- Absence of medical comorbidity, including obstructive sleep apnoea, body mass index (BMI) > 40 (because of the extended operation time and complexity of the procedure) and no history of acute coronary syndrome
- The knee does not require complex reconstruction by corrective osteotomy

If the patients agreed, they were enrolled for the same-stage, bilateral TKR procedure. If the patients disagreed, the reasons were recorded, and a two-stage, bilateral TKR procedure was performed.

**Fig. 1:**
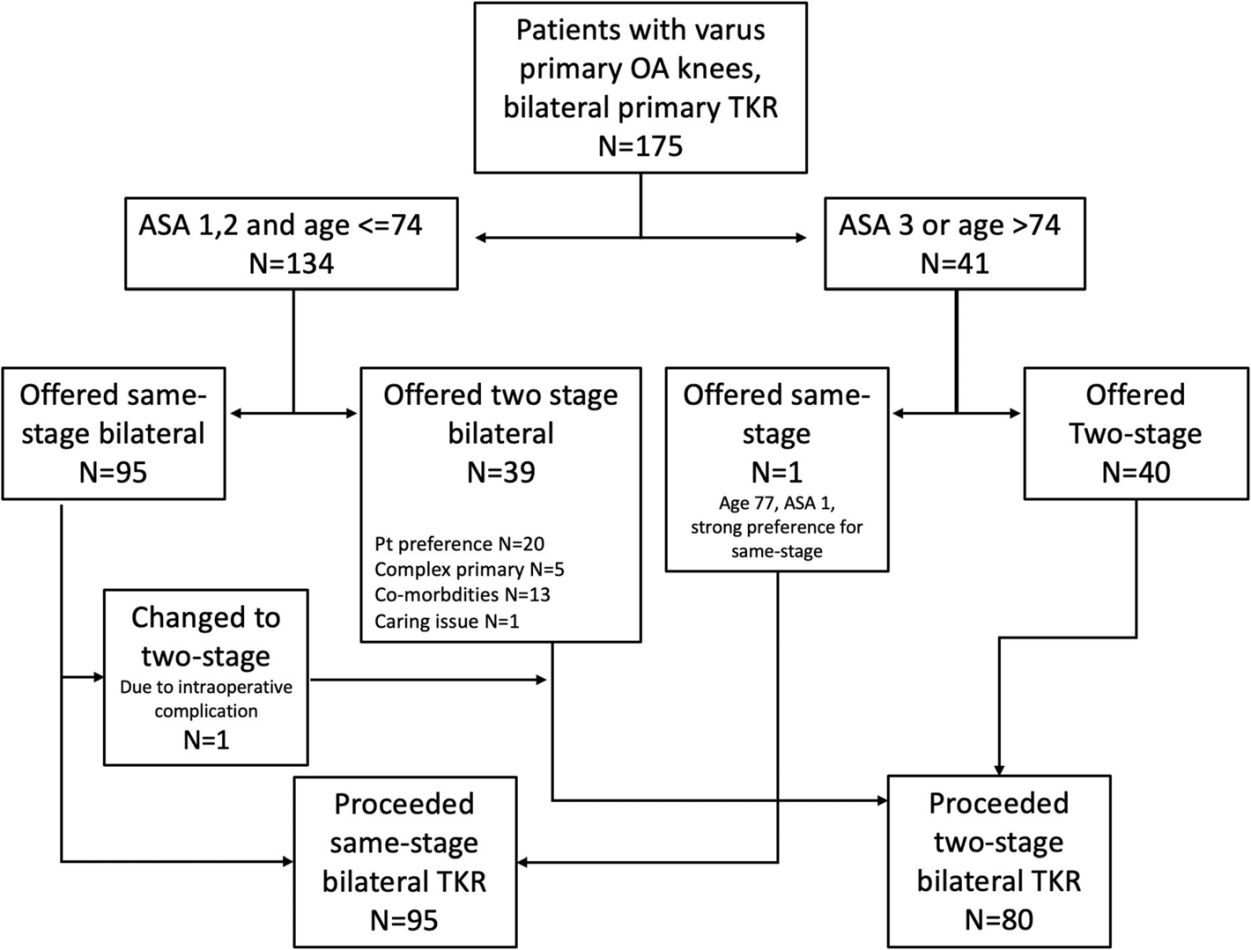
Consolidated Standards of Reporting Trials (CONSORT) diagram

We identified a total of 175 patients. The Consolidated Standards of Reporting Trials (CONSORT) flow diagram is shown in Fig. 1. Among them, 95 patients (26 male and 69 female) received a same-stage TKR and 80 patients (22 male, 58 female) received a two-stage, bilateral TKR. Twenty-two patients fit the inclusion criteria for the same-stage, bilateral TKR but were selected for the two-stage, bilateral TKR group for the following reasons: 20 (90.9%) patients preferred the two-stage procedure; one (4.5%) case was related to a caring plan issue; and one (4.5%) patient had initially been prepared for the same-stage, bilateral TKR, but the second knee surgery was abandoned intra-operatively due to an injury to the popliteal artery of the first knee.

Only one patient who did not fit the inclusion criteria, a 77-year-old man with good past health and having ASA 1, received the same-stage surgery; he expressed a very strong preference for same-stage, bilateral TKR.

Institutional Review Board ethics approval was obtained from the Chinese University of Hong Kong, Hong Kong SAR, CRE Ref. No. 2019.250 (Submission Ref. No. NTEC-2019-0093). Informed consent is not required as this is a retrospective review of medical records which contain no identifiable information of the patient.

### Perioperative protocol – ERAS protocol

#### Pre-operation

Before the operation, structured education classes were provided by JRC specialty nurses, physiotherapists, and occupational therapists. All patients participated in a 4r-week ‘pre-hab’ programme, organised by physiotherapists and occupational therapists. An appropriate anaesthetic plan and a post-operative analgesic regimen were tailor-made. Any medical comorbidity requiring workup and optimisation was settled during a pre-operative in-patient medical consultation. Almost all TKR patients were admitted on the same day of surgery, except those requiring bridging therapy for warfarin and those requiring pace-maker adjustments. Iron-deficiency anaemia with haemoglobin < 10.0 g/dL was treated by orally administering iron supplementation. JRC specialty nurses liaised with different parties to ensure fluent discharge process.

#### Intra-operatively

All TKR surgeries were performed by the same team of three senior specialist joint surgeons (FCH, KKB, HYW). The procedures were performed using tourniquets unless the distal pulses were not palpable, via the medial para-patella approach, with femoral computer navigation, and tibial extra-medullary guide. A variety of commercially available implant brands were used depending on availability and surgeon preferences. All prostheses were fixed with cement and tourniquet pressure was released for haemostasis. All patients received a peri-articular injection of a 100-ml cocktail (20 ml 0.5% levobupivacaine, 2 ml 1:10,000 adrenaline, 5 mg morphine, 30 mg ketorolac (in 1 ml) and normal saline up to 100 ml) before wound closure unless contraindicated (e.g. allergy or anaesthetically unfit). An intra-articular injection of 2 g of tranexamic acid was given. One suction drain was inserted only if the patient was taking double antiplatelet therapy or anticoagulants. Therefore, drains are always inserted in both knees if needed. In the case of same-stage, bilateral TKR, while one surgeon started closing the capsule of the first knee, another surgeon would start the incision on the other knee. Only one tourniquet was inflated at any time. Implant choice is decided by surgeons’ preference and implant availability. It is our practice to always uses same implant for both knees to prevent patients’ worry that one implant is better than the other.

#### Post-operation

All patients received multimodal orally administered analgesia. Pregabalin was started the day before TKR for 3 days. Paracetamol and etoricoxib were administered at post-operative day zero for 5 days. A weak orally administered opioid analgesic (tramadol) was administered for 3 days; it would be terminated early if patients complained of nausea and dizziness. Patient-controlled analgesia was prescribed by anaesthetists only when necessary and was typically limited to the first post-operative day.

Physiotherapists provided post-operative day-zero rehabilitation if the patient could tolerate it. In most cases, rehabilitation exercises were performed twice a day.

Orally administered aspirin (80 mg daily for 2 weeks) was administered for thromboembolism prophylaxis unless contraindicated. Blood transfusion was protocol-driven. For a haemoglobin level < 10 g/dL, orally administered iron supplement was given. Patients were transfused only when haemoglobin was < 8 g/dL or when they were symptomatic (including shortness of breath, tachycardia, palpitation, chest pain).

### Discharge and after discharge

Patient discharge was also protocol-driven. They were allowed to return home when physiotherapists documented stable, independent frame walking and when pain control was reasonable; this usually occurred at post-operative days 3 to 4.

A secure safety net that comprises JRC specialty nurses, physiotherapists, and occupational therapists was established during patient recovery at home. Outpatient physiotherapy was guaranteed on the third working day after discharge. Assistance from the JRC nursing clinic and from the JRC ward is available 24-hourly, and patients will not need to resort to emergency department attendance.

### Measurement of outcomes

The data sources included the JRC Registry in an Excel file continuously updated by JRC specialty nurses, the Clinical Management System (the universal electronic health record system in Hong Kong) utilised by the Hospital Authority, and the rehabilitation database maintained by physiotherapists and occupational therapists.

Demographic data, including age, gender, ASA grade, pre-operative Knee Society Knee Score (KS), Knee Society Function Score (FS), and Oxford Knee Score (OKS), was captured. The KS, FS, and OKS were obtained by pre-operative occupation therapy assessment. ASA grades were retrieved by pre-operation anaesthetist assessment. Age, gender, implant choices, and drain usage were obtained from patient records.

Cost-effectiveness data included average length of stay (ALOS), operating theatre time (OTT), number of post-operative physiotherapy sessions (PhyS), and number of occupational therapy sessions (OccS) was captured. ALOS was obtained from patient records. Operation theatre turnover time was obtained from the operation theatre’s record (automatically generated from anaesthetist’s electronic record) of last operation’s completion time (defined as completion of wound closure) to the next operation’s anaesthesia-ready time (defined as completion of spinal anaesthesia injection, or completion of intubation in general anaesthesia, and ready for surgeons to position the patient) was obtained. The operation theatre turnover time is defined as the interval between last operation’s completion time and the next operation’s anaesthesia ready time. We sampled 4 months, from July to September 2017, and obtained the mean turnover time from the operation theatre’s record. The cost of each day of hospital stay, physical therapy sessions and occupational therapy sessions is estimated by the gazetted public hospital charges on all non-entitled person (not covered by public welfare.

Operating theatre consumables include those used outside theatres (e.g. sterilisation and sterile packaging of re-usable instruments, autoclaves, etc.) and inside the theatre (e.g. personal protective gear, gloves, single-use drapes, etc.). The consumable cost is obtained from internal financial report for fiscal year 2017. The implant purchase cost per knee was equal for both groups.

Safety profile data included pre-operative haemoglobin (PreHb), haemoglobin level on the first day post-operation (HbD1), haemoglobin level drop between pre-operation and day 1 post-operation (HbDrop), transfusion percentage, transfusion volume (TV), surgical complication rate (SurgCR), medical complication rate (MedCR), and 30-day and 90-day unplanned readmission rate (UPR).

The physiotherapist assessed patients using standard and commonly used tools from the Fullerton Functional Fitness Test Battery including the Thirty-second Chair Stand Test (CST) [13], the Timed Up and Go Test (TUGT) [12], and the Numeric Pain Rating Scale (NPRS). The CST requires the patient to sit upright – that the patient sit in the middle of the chair, with crossed arms on the chest, keeps their feet flat and with a back straight. Then, upon the instruction word ‘Go’, the patient will rise to a straight standing position, then sit down to the starting position. The number of cycles that the patient can fully complete in 30 s is recorded; the more the better. The TUGT requires the patient to fully sit, stand up according to the therapist’s command, walk for 3 m, turn around, walk back to the chair and sit down. Walking aids can be used if needed. The time needed to complete the entire cycle is recorded; the lower this is the better the performance is. The NPRS involves asking the patient to rate their subjective pain score from 0 (no pain) to 10 (most severe pain).

Rehabilitation results were collected at different pre-operative and post-operative times as follows:

- ‘Pre-operation’: when patients finished ‘pre-hab’
- ‘D0 post-op’: when patients performed the first rehabilitation after the operation, typically on the same day post-operation if possible
- ‘D/C from PT’: when patients finished their final rehabilitation session and were discharged from outpatient physiotherapy.

### Data analysis

A two-tailed Student’s *T* test was used for comparing the two groups’ age, pre-operation KS, FS, OKS, ALOS, OTT, PhyS, OccS, PreHb, HbD1, HbDrop. *P* <  0.05 is considered statistically significant.

The chi-square test was used in comparing ASA grade, gender, implant choice, drain use, transfusion percentage, transfusion volume (TV), SurgCR, MedCR, and 30-day and 90-day UPR.

One-way analysis of variance (ANOVA) was used for CST, TUGT, and NPRS. Post-hoc subgroup analysis using a Bonferroni test was then used to compare the differences between the same-stage and first knee in the two-stage, between the same-stage and second knee in the two-stage, and between the first and second knee in the two-stage. In these analyses, *P* <  0.017 was statistically significant (Bonferroni corrected *P* value).

A ‘young and fit’ subgroup of two-stage patients that are similar to the same-stage group, namely non-obese, non-complex, age < 75 years, and ASA grade 1 or 2, was compared to the same-stage group to alleviate patient selection bias. The parameters that were compared included gender and number of patients, age, KS, FS, OKS, ALOS, transfusion percentage, TV, reoperation rate (RR), SurgCR, and MedCR.

SPSS 21.0 (IBM Corp., Armonk, NY, USA) was used for statistical analysis.

## Results

### Demographics

Their demographics and pre-operation function scores are listed in Table 1. The same-stage group had younger patients (65.6 (51–77, SD 5.31) vs 68.7 (56–79, SD 6.46), *P* = 0.041) with a ‘better’ ASA composition (same-stage ASA 1 5.26%, ASA 2 94.74%, ASA 3 0%, two-stage ASA 1 1.96%, ASA 2 67.97%, ASA 3 30.07%; *P* <  0.001). The same-stage group had similar FS and OKS (Table 1). For KS, the difference of mean value is small (same-stage 44.3 (18–79, SD 13.54, 95% confidence interval (CI) 41.6–47) vs two-stage 44.5 (12–77, SD 12.82, 95% CI 41.7–47.3), and is statistically insignificant as 95% CI overlap.

**Table 1:** Results of comparison between same-stage and two-stage total knee replacements (TKRs)

Parameters	Same-stage, bilateral TKR (*n* = 95)	Two-stage, bilateral TKR (*n* = 80)	*P*
Demographics			
Age (years)	65.6 (51–77, SD 5.31)	68.7 (56–79, SD 6.46)	**0.041**
Gender	26 M, 69 F	22 M, 58 F	0.894
ASA grade	1: 5, 5.26%,	1: 3, 3.75%	**< 0.001**
	2: 90, 94.74%	2: 54, 67.97%,	
	3: 0%	3: 24, 30.07%	
Implant choice	Triathlon 124 (65.3%) Attune 66 (34.7%)	Triathlon 112 (70.0%) Attune 48 (30.0%)	0.505
Pre-operation function			
FS	50.2 (5–80, SD 13.19,	47.3 (5–90, SD 16.00,	0.706
	95% CI 47.7–52.9)	95% CI 43.8–50.8)	
KS	44.3 (18–79, SD 13.54,	44.5 (12–77, SD 12.82,	**0.025**
	95% CI 41.6–47)	95% CI 41.7–47.3)	
OKS	24.9 (8–54, SD 6.69,	25.2 (6–54, SD 7.62,	0.855
	95% CI 23.5–26.3)	95% CI 23.5–26.9)	
Cost-effectiveness			
LOS (days)	6.64 (3–12, SD 2.14,	12.35^a^ (7–65, SD 6.67,	**< 0.001**
	95% CI 6.21–7.07)	95% CI 10.9–13.8)	
		6.07^b^ (2–43)	**< 0.001**
OTT^c^ (mins) for 2 knees	152.8^c^ (68–478, SD 31.34,	180.3^c^ (111–417^c^, SD 48.46,	**< 0.001**
	95% CI 147.1–159.2)	95% CI 170–191)	
PhyS	14.56^d^ (3–26, SD 4.78,	17.90^d^ (11–37, SD 4.68,	**< 0.001**
	95% CI 13.5–15.6)	95% CI 17.0–18.8)	
OccS	3.56^d^ (2–8, SD 1.97,	7.34^d^ (2–24, SD 2.40,	**< 0.001**
	95% CI 3.16–3.96)	95% CI 6.81–7.87)	
Safety			
PreHb (g/dL)	13.50 (9.8–16.6, SD 1.10,	13.00 (9.1–15.7, SD 1.31,	**0.002**
	95% CI 13.3–13.7)	95% CI 12.7–13.3)	
HbD1 (g/dL)	10.47 (7.0–14.1, SD 1.28,	10.93 (7.4–14.1, SD 1.43,	**0.003**
	95% CI 10.2–10.7)	95% CI 10.6–11.2)	
HbDrop (g/dL)	3.03 (0.0–6.0, SD 1.21,	2.07 (− 0.1–6.2, SD 1.01,	**< 0.001**
	95% CI 2.79–3.27)	95% CI 1.85–2.29)	
Transfusion percentage	8.42% (16 of 190 knees)	3.75% (6 of 160 knees)	0.078
TV (units) for 2 knees	0.18 (0–6, SD 0.45,	0.09 (0–4, SD 0.76,	0.116
	95% CI 0.090–0.27)	95% CI − 0.52–0.70)	
SurgCR	7.37% (7 of 95 operations)	5.63% (9 of 160 operations)	0.511
MedCR	3.68% (7 of 190 knees)	5.63% (9 of 160 knees)	0.386
	10.5% (10 of 95 operations)	6.20% (10 of 160 operations)	0.155
RR	3.16% (3 of 95 operation)	4.38% (7 of 160 operations)	0.548
30-days UPR	2.11% (2 of 95 operations)	6.92% (11 of 160 operations)	0.094
90-days UPR	4.21% (4 of 95 operations)	9.43% (15 of 160 operations)	0.129

*Abbreviations*: *ALOS* average length of stay, *ASA* American Society of Anesthesiologists, *FS* Knee Society Function Score, *HbD1* haemoglobin level on first day post-operation, *HbDrop* haemoglobin level drop, *KS* Knee Society Knee Score, *MedCR* medical complication rate, *OKS* Oxford Knee Score, *OccS* occupational therapy sessions, *OTT* operation theatre time, *PhyS* physical therapy sessions, *PreHb* pre-operative haemoglobin, *RR* reoperation rate, *SD* standard deviation, *SurgCR* surgical complication rate, *TV* transfusion volume, *UPR* unplanned readmission, *95% CI* 95% confidence interval

Statistically significant *P* values are presented in **bold**

^a^patient-specific total ALOS of 2 knees in an individual patient

^b^knee-specific ALOS of one knee

^c^presented as the total operation time for both knees

^d^presented as the total number for both knees

The same-stage TKR and a subgroup of two-stage TKR patients (‘young and fit’ subgroup) with ASA grades 1–2, age < 75 years, non-complex, and non-obese, similar to the same-stage group, showed no significant difference in gender composition and age in two groups. The two groups have no difference in FS, KS, and OKS (Table 2).

**Table 2:** Results of comparison between same-stage and two-stage ‘young and fit’ subgroup (non-obese, non-complex, ASA 1 or 2, age < 75 years)

Subgroup analysis demographics	Same-stage, bilateral TKR (*n* = 95)	Two-stage, bilateral TKR ‘young and fit’ subgroup (*n* = 22)	*P*
Gender and number	26 M, 69 F	6 M, 16 F	0.556
Age	65.6 (51–77, SD 5.31, 95% CI 64.5–66.7)	66.1 (56–74, SD 4.94, 95% CI 64–68.2)	0.415
ASA grade	1: 5, 5.26%,	1: 3, 13.64%	0.161
	2: 90, 94.74%	2: 19, 86.36%	
	3: 0, 0%	3: 0, 0%	
FS	50.3 (5–80, SD 12.99,	50.1 (5–90, SD 18.64,	0.083
	95% CI 47.7–52.9)	95% CI 42.3–57.9)	
KS	44.3 (18–79, SD 13.54,	44.7 (12–74, SD 12.97,	0.412
	95% CI 41.6–47)	95% CI 39.3–50.1)	
OKS	24.9 (8–54, SD 6.69,	25.3 (6–54, SD 8.41,	0.556
	95% CI 23.5–26.3)	95% CI 21.5–28.5)	
ALOS (Days)	6.64 (3–12, SD 2.13,	11.29^a^ (7–15, SD 1.55,	**< 0.001**
	95% CI 6.21–7.07)	95% CI 10.6–11.9)	
HbDrop (g/dL)	3.03 (0.0–6.0, SD 1.21,	2.20 (**−** 0.1–6.2, SD 1.02,	**< 0.001**
	95% CI 2.19–3.27)	95% CI 1.77–2.63)	
TV (units) for 2 knees	0.18 (0–6, SD 1.21,	0.10 (0–4, SD 1.02,	0.289
	95% CI − 0.063–0.42)	95% CI − 0.36–0.53)	
Transfusion percentage	8.42% (16 of 190 knees)	9.09% (4 of 44 knees)	0.886
RR	3.16% (6 of 190 knees)	6.81% (3 of 44 knees)	0.255
Surg CR	3.68% (7 of 190 knees)	9.09% (4 in 44 knees)	0.127
Med CR	10.5% (10 of 95 operations)	4.55% (2 of 44 operations)	0.243

*Abbreviations*: *ALOS* average length of stay, *ASA* American Society of Anesthesiologists, *FS* Knee Society Function Score, *HbD1* haemoglobin level on first day post-operation, *HbDrop* haemoglobin level drop, *KS* Knee Society Knee Score, *MedCR* medical complication rate, *OKS* Oxford Knee Score, *OccS* occupational therapy sessions, *OTT* operation theatre time, *PhyS* physical therapy sessions, *PreHb* pre-operative haemoglobin, *RR* reoperation rate, *SD* standard deviation, *SurgCR* surgical complication rate, *TV* transfusion volume, *UPR* unplanned readmission, *95% CI* 95% confidence interval

Statistically significant *P* values are presented in **bold**

^a^patient-specific total ALOS of 2 knees in an individual patient

^b^knee-specific ALOS of one knee

^c^presented as the total operation time for both knees

^d^presented as the total number for both knees

Tourniquets were used for all cases. Drains were inserted in four knees (from two patients) from the two-stage group and none in the same-stage group. No drains were used in the two-stage ‘young and fit’ subgroup. There was no statistically significant difference (*P* = 0.062). The implants used were all posterior stablising, including the Triathlon Knee System (Stryker, Kalamazoo, MI, USA) and the Attunes Knee System (DePuy Synthes, West Chester, PA, USA) (Table 1). There was no significant difference in implant choices between two groups.

### Cost comparison

The key information regarding cost comparison is listed in Table 1.

During the study period, Hong Kong public hospitals charged each non-entitled person HK$4680 per day of inpatient stays and HK$1110 per specialist clinic visit, occupational therapy, and physiotherapy clinic session, which were assumed to be the unit cost. According to our centre’s Sterile Supply Unit data for the fiscal year 2017, HK$2421 (US$312.3) was saved per same-stage, bilateral TKR procedure.

For each same-stage, bilateral TKR case performed instead of two-stage, bilateral TKR, the following resources were spared:

- 5.71 days of ALOS (estimated saving HK$26,722.8)
- 3.34 (or 18.6%) physiotherapy sessions (estimated saving HK$11,733)
- 3.78 (or 51.5%) occupational therapy sessions (estimated saving HK$4418)
- One set of operating theatre consumables (HK$2421)
- 57.9 min (34–149) of operation theatre turnover time
- 27.4 min of operation theatre time

By considering these figures, we estimated that a total of HK$45,295 or US$5822 (US$1 = HK$7.78) could be saved for every patient who underwent same-stage, bilateral TKR rather than two-stage, bilateral TKR. This estimated a cost saving of 27.6% per TKR.

When comparing same-stage group with the two-stage ‘young and fit’ subgroup, a 4.65 days’ shorter average length of stay (ALOS) was saved (Table 2).

### Safety profile

The same-stage group initially had a slightly better blood haemoglobin level (same-stage 13.50 g/dL, (9.8–16.6, SD 1.10, 95% CI 13.3–13.7) vs two-stage 13.00 g/dL (9.1–15.7, SD 1.31, 95% CI 12.7–13.3); *P* = 0.002) but experienced a haemoglobin drop 0.96 g/dL more than the two-stage group (same-stage 3.03 (0.0–6.0, SD 1.21, 95% CI 2.79–3.27) vs two-stage 2.07 (− 0.1–6.2, SD 1.01, 95% CI 1.85–2.29); *P* <  0.001). However, this difference did not translate into a higher transfusion volume (same-stage 0.18 (0–6, SD 0.45, 95% CI 0.090–0.27) vs two-stage 0.09 (0–4, SD 0.76, 95% CI − 0.52–0.70); *P* = 0.116) or a higher transfusion rate (same-stage 8.42% (16 of 190 knees) vs two-stage 3.75% (6 of 160 knees); *P* = 0.078). There were no statistically significant differences in SurgCR, MedCR, RR, 30-day UPR or 90-day UPR (Table 1). HbD1 has no statistically significant difference as 95% CIs overlap (same-stage 10.47 (7.0–14.1, SD 1.28, 95% CI 10.2–10.7) vs two-stage 10.93 (7.4–14.1, SD 1.43, 95% CI 10.6–11.2)]. All transfusion occurred with Hb < 8.0 g/dL.

The causes of reoperation and surgical and medical complications are listed in Table 3. There were no cardiovascular events, pulmonary embolism or mortality in either group.

**Table 3:** Reoperation causes, surgical and medical complications

Complications and reoperation causes	Same-stage, bilateral TKR	Two-stage, bilateral TKR
Reoperation causes		
Persistent wound discharge requiring irrigation and debridement	2	4
Wound haematoma requiring open drainage	1	1
Periprosthetic infection		1
Intra-articular haematoma		1
Surgical complications		
*Reoperation causes above-mentioned*	3	7
Medial collateral ligament injury/avulsion	2	
Patella-tendon insertion avulsion	1	
Intra-operative fracture	1	2
Popliteal artery laceration		1
Medical complications		
Ischaemic stroke	1	
Chest infection	4	
Urinary tract infection	2	4
Acute renal failure		1
Insomnia	1	
Gastrointestinal bleeding	1	
Deep vein thrombosis	1	
Congestive heart failure		1
Aspirin-induced gastrointestinal upset		1
Transient fast atrial fibrillation		1
Transient hypotension (resolved with only fluid replacement)		1
Common peroneal nerve palsy		1

*TKR* total knee replacement

We also compared the same-stage TKR and a subgroup of two-stage TKR patients (‘young and fit’ subgroup) with ASA 1–2, age < 75 years, non-complex, and non-obese in the two-stage group. The results are listed in Tables 2 and 4. HbDrop was 0.83 g/dL more in the same-stage group (same-stage 3.03 (0.0–6.0, SD 1.21, 95% CI 2.19–3.27) vs two-stage 2.20 (**−** 0.1–6.2, SD 1.02, 95% CI 1.77–2.63); *P* <  0.001), but this is statistically insignificant as 95% CIs overlap. There was no statistically significant difference in transfusion volume (TV), transfusion percentage, RR, SurgCR or MedCR (Table 2).

**Table 4:** Reoperation causes, medical and surgical complications of two-stage, bilateral TKR ‘young and fit ‘subgroup with ASA 1 or 2, age < 75 years , non-complex, non-obese

Complications and reoperation causes	Two-stage, bilateral TKR ‘young and fit’ subgroup
Reoperation causes	
Persistent wound discharge requiring irrigation and debridement	3
Surgical complications	
*Reoperation causes above-mentioned*	3
Popliteal artery laceration	1
Medical complications	
Transient fast atrial fibrillation	1
Urinary tract infection	1

### Rehabilitation

**Fig. 2:**
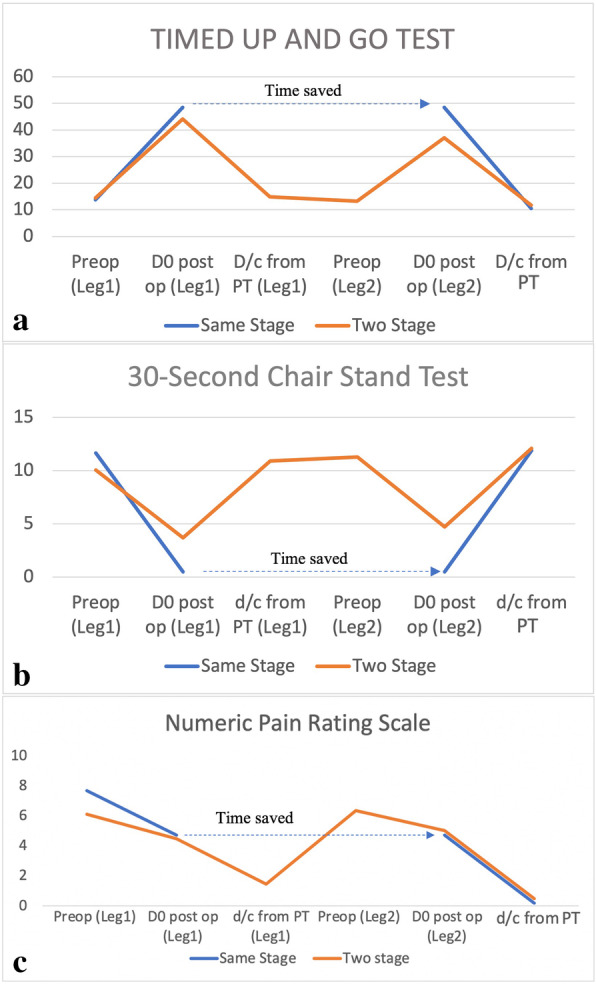
**a** Timed Up and Go Test (TUGT): results are presented in seconds. **b** Thirty-second Chair Stand Test (CST): results are presented as number of cycles. **c** Numeric pain rating scale (NPRS). Pre-op (Leg 1): when patients were discharged from pre-operative physiotherapy. D0 post-op (leg 1): at the first post-operative rehabilitation, in the same-stage group and the first knee in two-stage group. D/C from PT (leg 1): when patient was discharged from physiotherapy for the first knee in the two-stage group. Pre-op (leg 2): when patient was discharged from pre-operative physiotherapy for the second knee in two-stage group. This time point did not exist for the same-stage group. D0 post-op (leg 2): the first post-operative rehabilitation in the second knee in the two-stage group. For the same-stage group, this time point is the same as D0 post-op (leg 1). D/C from PT the moment when the patient was discharged from physiotherapy (PT) at the end of all rehabilitation. The dotted line with the arrow indicates the time spared in same-stage, bilateral total knee replacement (TKR) group

The rehabilitation results are listed in Fig. 2 and Table 5 and demonstrate rehabilitation outcomes at different times between the same-stage and two-stage, bilateral TKR.

**Table 5:** Details for the (a) Timed Up and Go Test (TUGT) (b) 30-s Chair Stand Test (CST), (c) Numeric Pain Rating Scale (NPRS)

**TUGT**	Pre-operation (in seconds)	D0 post-op (in seconds)	D/C from PT (in seconds)
Same-stage	13.78 (SD 8.09)	48.45 (SD 17.48)	10.57 (SD 2.78)
Two-stage 1st knee	14.40 (SD 5.77)	44.14 (SD 21.23)	14.91 (SD 5.19)
Two-stage 2nd knee	13.28 (SD 6.10)	37.02 (SD 24.45)	11.73 (SD 3.02)
ANOVA^a^ *P* value	0.112	**0.005**	**0.003**
Post-hoc Bonferroni test^b^			
Same-stage vs 1st knee	–	0.437	**< 0.001**
Same-stage vs 2nd knee	–	**0.003**	**0.003**
1st knee vs 2nd knee		0.141	**< 0.001**
**CST**	Pre-operation (no. of cycles)	D0 post-op (no. of cycles)	D/C from PT (no. of cycles)
Same-stage	11.64 (SD 4.72)	0.48 (SD 1.52)	11.86 (SD 3.27)
Two-stage 1st knee	10.03 (SD 3.20)	3.69 (SD 3.71)	10.88 (SD 3.06)
Two-stage 2nd knee	11.28 (SD 2.79)	4.69 (SD 4.00)	12.08 (SD 2.96)
ANOVA^a^ *P* value	**0.006**	**< 0.001**	0.067
Post-hoc Bonferroni test^b^			
Same-stage vs 1st knee	**0.001**	**< 0.001**	–
Same-stage vs 2nd knee	0.344	**0.001**	–
1st knee vs 2nd knee	0.297	0.289	–
**NPRS**	Pre-operation (score 0–10)	D0 post-op (score 0–10)	D/C from PT (score 0–10)
Same-stage	7.69 (SD 1.48)	4.74 (SD 1.60)	0.19 (SD 0.83)
Two-stage 1st knee	6.08 (SD 1.85)	4.46 (SD 2.07)	1.44 (SD 1.51)
Two-stage 2nd knee	6.34 (SD 2.15)	5.00 (SD 1.88)	0.48 (SD 0.97)
ANOVA^a^ *P* value	**< 0.001**	**0.261**	**< 0.001**
Post-hoc Bonferroni test^b^			
Same-stage vs 1st knee	**< 0.001**	–	**0.001**
Same-stage vs 2nd knee	**0.001**	–	**0.003**
1st knee vs 2nd knee	0.820	–	**< 0.001**

*Abbreviations*: *ANOVA* analysis of variance, *D0 post-op* the first post-operative rehabilitation results in the second knee in two-stage group, D/C from PT the moment when the patient was discharged from physiotherapy (PT) at the end of all rehabilitation

^a^One-way ANOVA

^b^Post-hoc Bonferroni *P* value correction: significant if *P* < 0.0170

Before operation, the same-stage group performed similarly in the TUGT and slightly better in the CST (same-stage 11.64 (SD 4.72) vs two-stage first knee 10.03 (SD 3.20), two-stage second knee 11.28 (SD 2.79), *P* = 0.006). The same-stage group experienced a higher pain level (NPRS) before surgery (same-stage 7.69 (SD 1.48), two-stage first knee 6.08 (SD 1.85), two-stage second knee 6.34 (SD 2.15), *P* <  0.001).

During the first rehabilitation post-operation, same-stage patients performed worse in the Thirty-second Chair Stand Test (same-stage 0.48 (SD 1.52) vs two-stage first knee 3.69 (SD 3.71), two-stage second knee 4.69 (SD 4.00), *P* = < 0.001), and worse in the TUGT (same-stage 48.45 (SD 17.48) vs two-stage first knee 44.14 (SD 21.23), two-stage second knee 37.02 (SD 24.45), *P* = 0.005). However, the for pain level there is no difference for the two-stage procedure regardless of whether for the first or second knee [same-stage 4.74 (SD 1.60) vs two-stage first knee 4.46 (SD 2.07), two-stage second knee 5.00 (SD 1.88), *P* = 2.61).

At the final rehabilitation post-operation, same-stage patients performed better than two-stage patients in the TUGT (same-stage 10.57 (SD 2.78) vs two-stage first knee 14.91 (SD 5.19), two-stage second knee 11.73 (SD 3.02), *P* = 0.003), they performed with no difference in the Thirty-second Chair Stand Test (same-stage 11.86 (SD 3.27) vs two-stage first knee 10.88 (SD 3.06), two-stage second knee 12.08 (SD 2.96), *P* = 0.067), and reported lower pain levels (same-stage 0.19 (SD 0.83) vs two-stage first knee 1.44 (SD 1.51), two-stage second knee 0.48 (SD 0.97), *P* <  0.001).

Comparison is made among two-stage patients for the first and second knee by post-hoc analysis. At the end of rehabilitation, the second knee performed better than the first knee in the TUGT (14.91 (SD 5.19) vs 11.73 (SD 3.02), *P* <  0.001) but no difference in the Thirty-second Chair Stand Test (10.88 (SD 3.06) vs 12.08 (SD 2.96), *P* = 0.067). The pain level has improved (first knee 1.44 (SD 1.51) vs second knee 0.48 (SD 0.97), *P* <  0.001).

## Discussion

We have demonstrated that same-stage, bilateral TKR can reduce cost without significant compromise on safety profile and rehabilitation performance. There was no significant difference in surgical complication rate, medical complication rate, readmission rate, and 30-day and 90-day unplanned readmission rate. The haemoglobin drop difference is narrow although statistically significant (less than 1 g/dL), and unlikely clinically significant with no difference in transfusion volume or transfusion rate. The safety profile comparison is not confounded by same-stage group’s better ASA grading, younger age, non-complexity, and non-obesity, according to a subgroup analysis of the ‘young and fit’ subgroup. The haemoglobin drop difference is statistically insignificant as 95% CIs overlap, and the difference in mean value is narrow (0.83 g/dL). There is no difference in transfusion rate or transfusion volume (Table 2).

Our results of about 27% saving of cost are comparable to those of Reuben et al., who reported a 36% reduction in cost for same-stage compared to two-stage TKR procedures [14]. For each same-stage, bilateral TKR patient, a total of 85.3 min could be spared (operation theatre time of 27.4 min and turnover time of 57.9 min). Assuming a theatre operates from 8 am to 5 pm (9 h) for elective cases, this means a 15.8% increase in efficiency, or one more case can be performed for every 6.33 cases. This factor is not quantifiable in terms of money. The patient load is large, and even small efforts to improve cost-effectiveness will make a huge cumulative difference.

Conflicting reports on the cost-effectiveness of same-stage versus two-stage, bilateral TKR can be explained by different settings, practices, and remuneration algorithms, among other factors [13]. Recently, Philips et al. reported that same-stage, bilateral TKR had lower per-episode inpatient cost but the same 90-day episode-specific cost as two-stage, bilateral TKR [5]. The reported cause was a higher outpatient rehabilitation facility cost, which neutralised the shorter inpatient rehabilitation. This study is based in a North America setting. In Hong Kong there is no outpatient rehabilitation facilities and patients are discharged when fit to stay at home. The unplanned readmission rate was non-inferior in our same-stage, bilateral TKR patients. Our ERAS protocol contributes significantly to the shorter length of stay.

Besides cost, our safety profile for same-stage surgery was similar—or even better—compared to two-stage surgery in all parameters. Similarly, a systemic review by Malahias et al. [15] reviewed 19 articles that concurred with other studies [16, 17] that mortality, revision rate, complication rates, thromboembolic events, and cardiac complications were at least similar or even improved for same-stage compared to two-stage TKR patients.

Few studies that have compared same-stage versus two-stage, bilateral TKR have separately compared the two-stage, bilateral TKR younger and fitter patient subgroup. This endeavour resulted in selection bias because healthier patients were selected for same-stage, bilateral TKRs in most cases, confounding the advantageous findings of same-stage, bilateral TKR. Our study alleviated this bias by separately comparing the two-stage, bilateral TKR subgroup with ASA 1 or 2, age < 75 years, non-obese, and non-complex. This subgroup comparison produced the same results as a the general comparison of the same-stage versus the two-stage TKR. This result is more convincing in demonstrating that same-stage, bilateral TKR is safe and not confounded by selection bias.

Compared to other studies, our same-stage, bilateral TKR group had a significantly lower transfusion percentage than other studies, including one recent study that reported rates of 15.8% for same-stage TKRs and 6.2% for two-stage TKRs [11]. This difference may suggest that our protocol, including refraining from drain insertion and iron-therapy for anaemia > 8 g/dL, is useful. Being conservative in terms of blood transfusion did not cause any cardiopulmonary incidents in our study.

Although increasing evidence has demonstrated the safety of same-stage, bilateral TKRs, including this study, current overriding opinions are still conservative in recommending same-stage, bilateral TKRs to patients [13], especially to patients with cardiopulmonary complications [18]. We believe that this study clearly defines a safe and well-proven perioperative pathway and patient selection criteria that other centres can follow and then reproduce our optimal results, so that appropriate patients are not excluded from same-stage, bilateral TKRs merely due to unnecessary safety concern.

Despite the same-stage operation having a non-inferior safety profile, some patients refuse same-stage because of worry of pain and poor walking performance. In this study, 40.8% of patients fit the same-stage operation but chose a two-stage operation due to their subjective wish. Our study can further alleviate pain and rehabilitation concern.

Some patients worry about poor performance after both knee surgery types, and think that they would perform better and have less pain if the operating was performed one knee at a time. Some patients may think that pain after the first knee in a two-stage operation is better than same-stage operation. Our results showed the contrary. In fact in the literature, studies have compared final functional results after same-stage versus two-stage, bilateral TKRs. However, to our knowledge, few studies have compared each time point in detail, particularly using the results after the first knee replacement in a two-stage, bilateral TKR. This is the merit of our study. We showed that same-stage surgery has better performance compared to the two-stage first knee operation in the TUGT while there was no difference in the Thirty-second Chair Stand Test. Pain level is better in same-stage surgery than in two-stage first knee surgery. Same-stage surgery is at least non-inferior to two-stage first knee in clinical outcomes.

By the end of rehabilitation, the same-stage group performed better in the TUGT and there was no difference in the Thirty-second Chair Stand Test. It showed that for same-stage surgery patients could enjoy a *better,* or at least *non-inferior*, functional outcome sooner than two-stage patients (sooner because only one surgery instead of two surgeries are required). This outcome is especially important in a locality where the waiting period for any knee replacement in the public healthcare system is long. A systemic review by Malahias et al. also revealed similar findings [15], that same-stage, bilateral TKR show improvement in some parameters and similar in others. This result could be due to the absence of painful knees that have not been operated on in same-stage patients, and that in two-stage, bilateral TKRs, the flexion contracture of the knee that has not been operated on will hinder the function of the operated knee [19]. Selection bias (selecting ‘better’ patients as in our study) may also underlie this finding.

For pain level, we found that the pain level in the same-stage group was similar to the two-stage group, immediately post-operation or when discharged from physiotherapy, even though the baseline pain level for the same-stage group was worse. This result is likely due to the multimodal analgesia administered intra-operatively and post-operatively according to our protocol. Similarly, Bagsby et al. reviewed 697 knees subjected to same-stage, bilateral TKR or unilateral TKR and found no difference in the pain level [20]. This finding may significantly affect patients’ decision-making and encourage them to choose same-stage, bilateral TKRs, because some patients avoid same-stage surgery due to worry of worse pain.

An age threshold of 75 years was chosen. The Literature found an increased surgical risk and anaesthetic risk for patients age older than 75 years [21–23]. Other thresholds including 80 years [24] or 85 years [25] were studied, but the benefit may be limited by a patient’s life expectancy. Thresholds of 70 were proposed [26], but this narrow indication may unnecessarily deprive suitable patients from the benefit of same-stage, bilateral TKR.

### Limitations

This study only represents a single centre’s experience with a limited sample size relative to other large registry data analyses. However, many outcomes, e.g. cost, transfusion percentage, thromboembolism prevalence, etc. depend heavily on perioperative practices, e.g. our centre’s unique ERAS protocol. It is the outcome of the entire patient management pathway rather than a surgery strategy itself that is compared. The fact that this is a single-centre study ensured homogeneity in patient selection and perioperative routines, including our ERAS protocol, which avoided confounding factors in larger, multi-centre studies. Only two patients (2.5%) from the two-stage group received drain insertion, and this number is unlikely to cause significant differences.

## Conclusion

This study demonstrates that same-stage, bilateral TKR can reduce healthcare costs, with non-inferior safety profiles and rehabilitation outcomes. The prerequisite is a suitable patient selection and perioperative protocol. Same-stage, bilateral TKRs can be recommended to patients who fulfil the selection criteria and could benefit patients and the entire healthcare system.

## Data Availability

The dataset analysed during the current study is available from the corresponding author on reasonable request.
